# Supplementary material for: Non-trivial stimuli-responsive collective behaviours emerging from microscopic dynamic complexity in supramolecular polymer systems
Source: Nat Commun. 2025 May 30;16:5030. doi: 10.1038/s41467-025-60150-4 (PMC12125348; doi:10.1038/s41467-025-60150-4)
Supplement: Supplementary file 1 — Supplementary Information [file 41467_2025_60150_MOESM1_ESM.pdf]

Supplementary Information: Non-Trivial Stimuli-Responsive Collective Behaviours Emerging from Microscopic Dynamic Complexity in Supramolecular Polymer Systems

Martina Crippa[1], Claudio Perego[2,*], and Giovanni M. Pavan[1,2,*]

[1]Department of Applied Science and Technology, Politecnico di Torino, Corso Duca degli Abruzzi 24, 10129 Torino, Italy
[2]Department of Innovative Technologies, University of Applied Sciences and Arts of Southern Switzerland, Polo Universitario Lugano, Campus Est, Via la Santa 1, 6962 Lugano-Viganello, Switzerland
[*]corresponding authors: Giovanni M. Pavan (giovanni.pavan@polito.it), Claudio Perego (claudio.perego@supsi.ch)

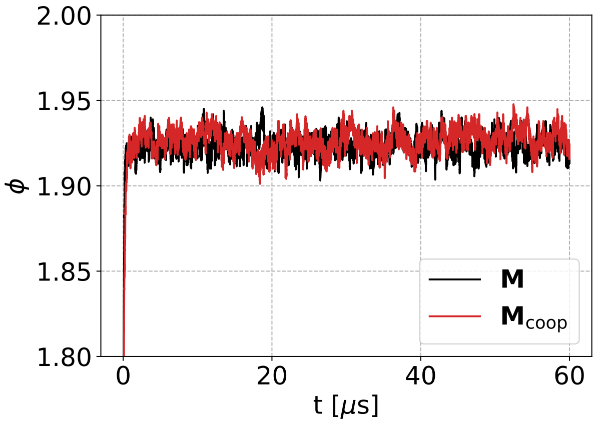

Figure S1: Average coordination number ϕ of $\mathbf{M}$ (black) and $\mathbf{M}_{\mathrm{coop}}$ systems starting from a configuration with randomly dispersed monomers, along 60 µs of trajectory. The equilibrium is reached during the first µss of dynamics.

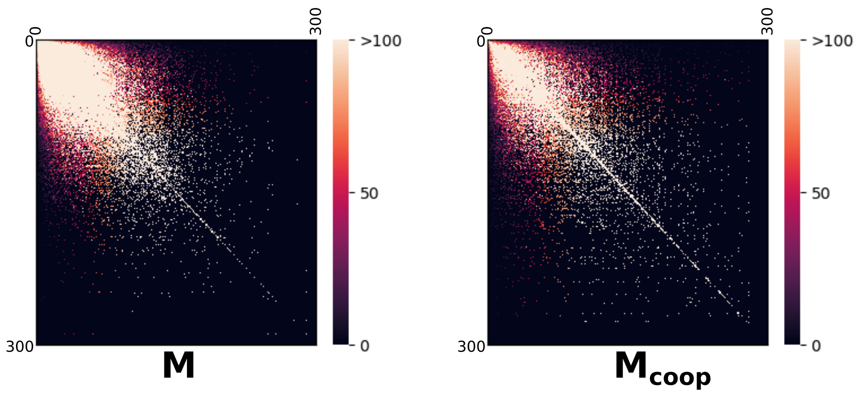

Figure S2: Transition matrices of $\mathbf{M}$ and $\mathbf{M}_{\mathrm{coop}}$ systems (the coloring of the matrix cells mirrors the entry values).

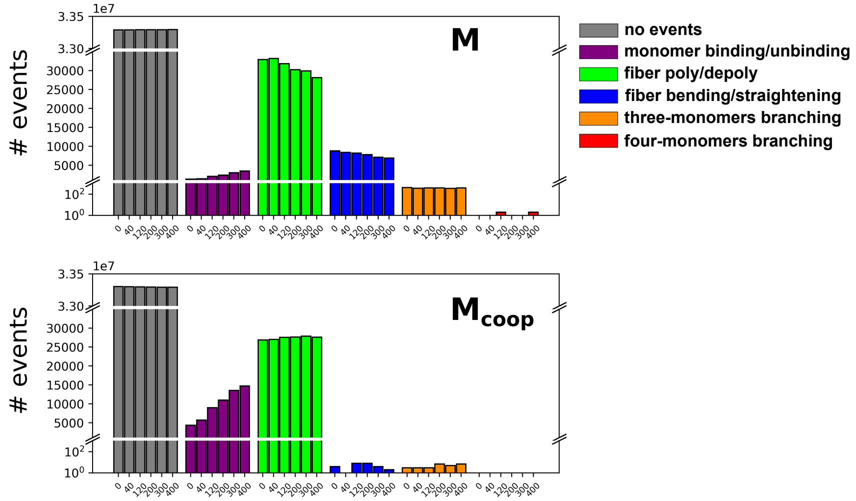

Figure S3: LENS mechanisms for the unperturbed and perturbed $\mathbf{M}$ and $\mathbf{M}_{\mathrm{coop}}$ systems, top and bottm respectively. In gray are colored the bare fiber monomers that do not change their neighborhood, in purple a single monomer binding/unbinding to another one (the latter being either part of an aggregate or not), green identifies polymerization and depolymerization of fibers, blue identifies non-neighboring monomers of the same fiber that either form or break a contact (fiber bending/straightening), orange is the formation (or elimination) of a branch, which is usually not stable, red is a rare and ephemeral event of branching (and un-branching) involving four centers.

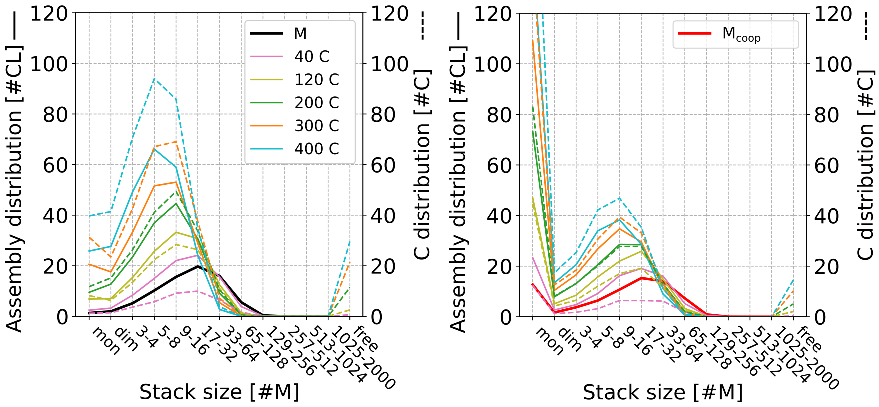

Figure S4: Average number of assemblies of a specific size (grouped in a binary log scale) per frame for both **M** and $\mathbf{M}_{\text{coop}}$ systems (left and right, respectively). The **C** distribution, *i.e.* the number of C attached to an assembly of a certain size, is also shown (dashed lines).

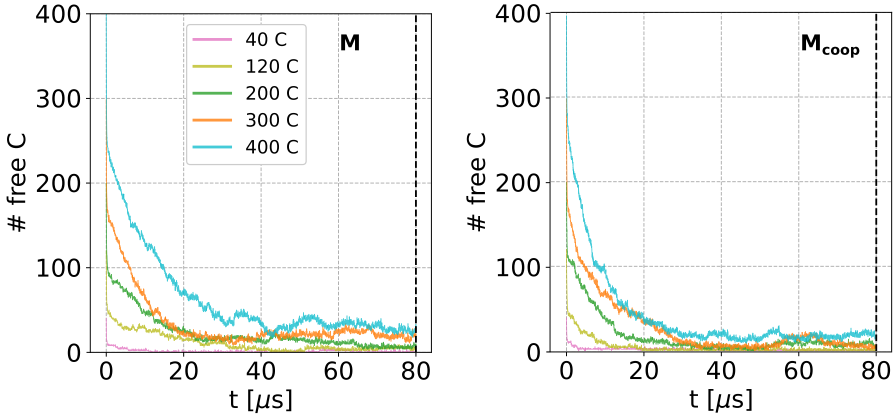

Figure S5: Number of free (not bound to another monomer or assembly) **C**s in perturbed **M** and $\mathbf{M}_{\text{coop}}$ systems. The initial t=80 µs of CG-MD from the insertion of the **C** are here considered.

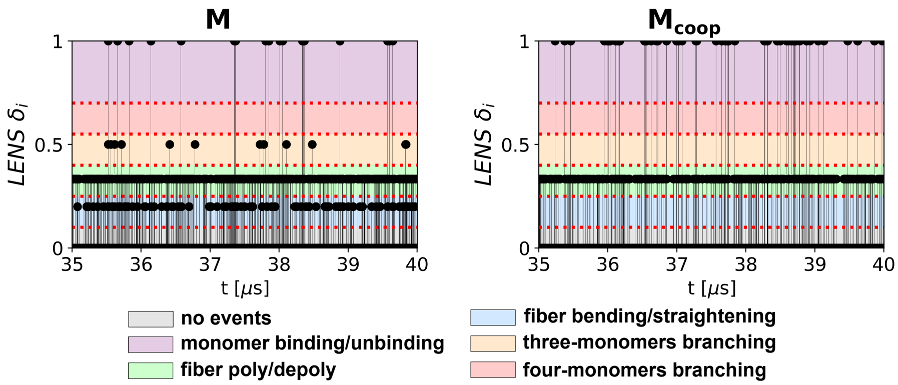

Figure S6: Example of LENS time-series. The $\mathbf{M}$ and $\mathbf{M}_{\text{coop}}$ systems without perturbation are reported as examples of LENS time series (last 5 µs of each MD simulation, representative of the equilibrium), with six different discrete values colored accordingly to the six communication mechanisms. The time-series are shown every 50 monomers for clarity.

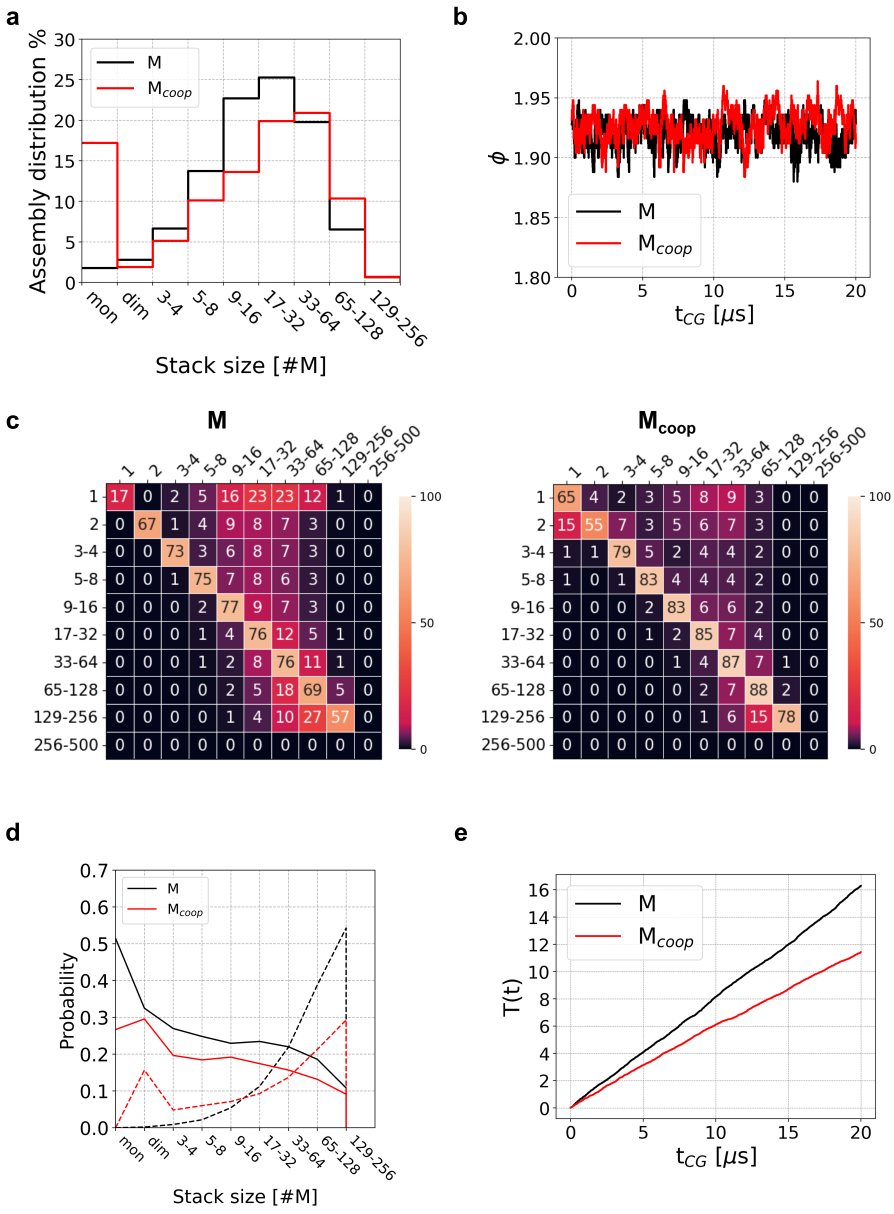

Figure S7: 500 monomers systems. (a) Assembly-size distribution (as % of the average number of assemblies), for the $\mathbf{M}^{500}$ (black) and $\mathbf{M}^{500}_{\text{coop}}$ (red) models, the sizes are grouped in log binary scale. (b) Average coordination ϕ of the $\mathbf{M}^{500}$ and $\mathbf{M}^{500}_{\text{coop}}$ systems. (c) Transition probability matrices (with $\Delta\tau$=15 ns) of $\mathbf{M}^{500}$ and $\mathbf{M}^{500}_{\text{coop}}$ systems. (d) Polymerization/depolymerization ratio as function of aggregate size (computed with $\Delta\tau$ = 15 ns). (e) Molecular traffic $T(t)$ of $\mathbf{M}^{500}$ and $\mathbf{M}^{500}_{\text{coop}}$ systems at the equilibrium.

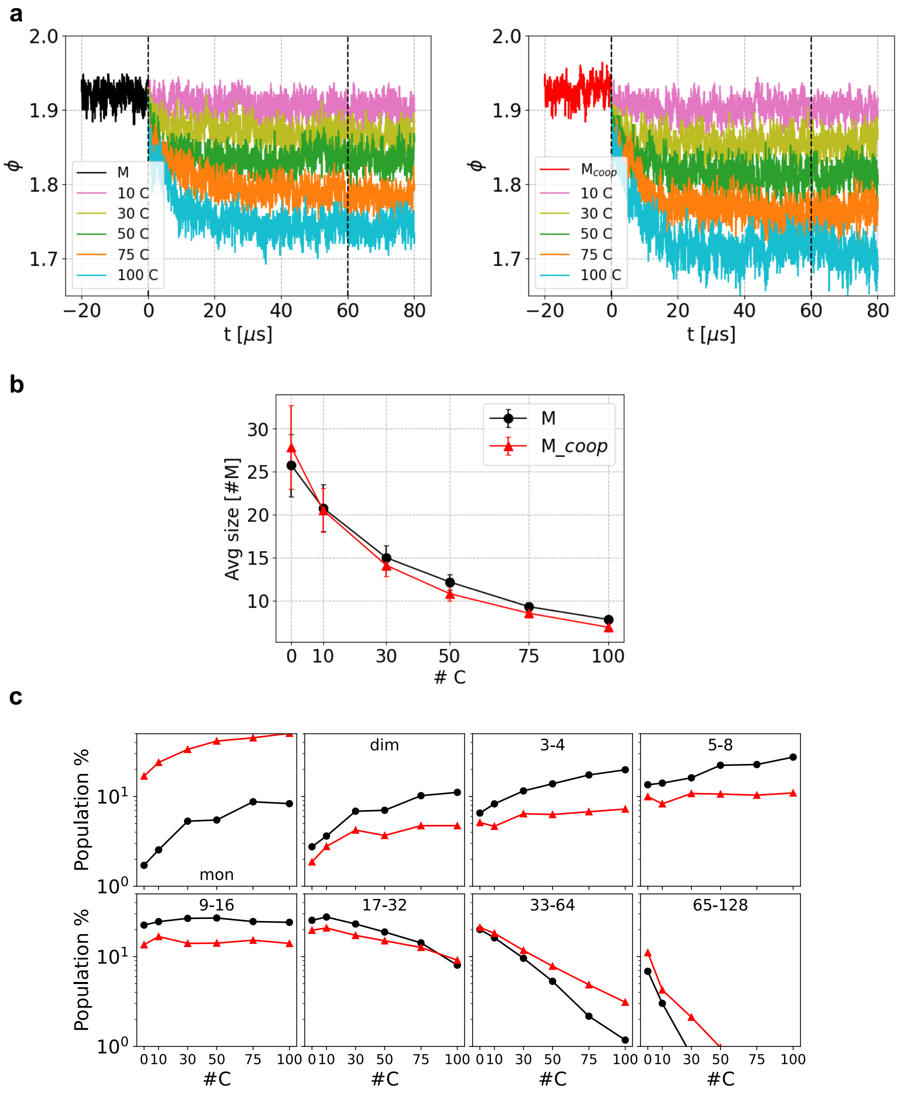

Figure S8: 500 monomers systems. (a) Average coordination of $\mathbf{M}^{500}$ (left) and $\mathbf{M}^{500}_{\text{coop}}$ (right) upon chain-stopper insertion. The unperturbed equilibrium behavior ($t < 0$) is affected at $t = 0$ with the insertion of different amounts of $\mathbf{C}$ (see legend). At $t = 60$ µs the systems can be safely considered in equilibrium with the added $\mathbf{C}$. (b) Average size at the equilibrium as a function of the number of inserted $\mathbf{C}$. Error bars show the standard deviation of the average size. (c) Equilibrium prevalence of different assembly sizes (in % of monomer content) as a function of the $\mathbf{C}$ number. For simplicity assembly sizes are grouped logarithmically.

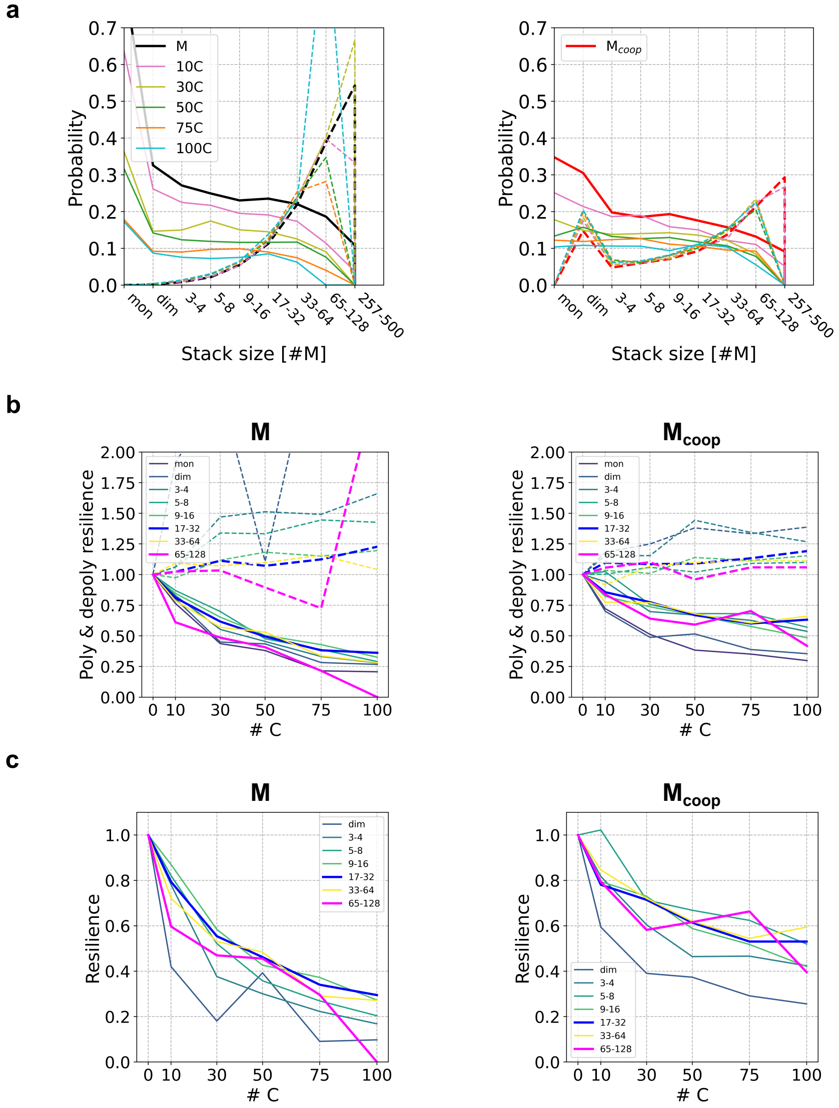

Figure S9: 500 monomers systems. (a) P_{poly} (solid) and P_{depoly} (dashed) (with $\Delta\tau$=15ns) as a function of the aggregate size, $\mathbf{M}^{500}$ (left) and $\mathbf{M}^{500}_{\text{coop}}$ (right) systems in equilibrium with different amounts of $\mathbf{C}$. (b) Relative probabilities of polymerization (solid) and depolymerization (dashed) of assemblies of different size-ranges, as a function of the $\mathbf{C}$ content, for $\mathbf{M}^{500}$ (left) and $\mathbf{M}^{500}_{\text{coop}}$ (right) systems. (c) *Resilience* of assemblies of different size-ranges, as a function of the $\mathbf{C}$ content, for $\mathbf{M}^{500}$ (left) and $\mathbf{M}^{500}_{\text{coop}}$ (right) systems.

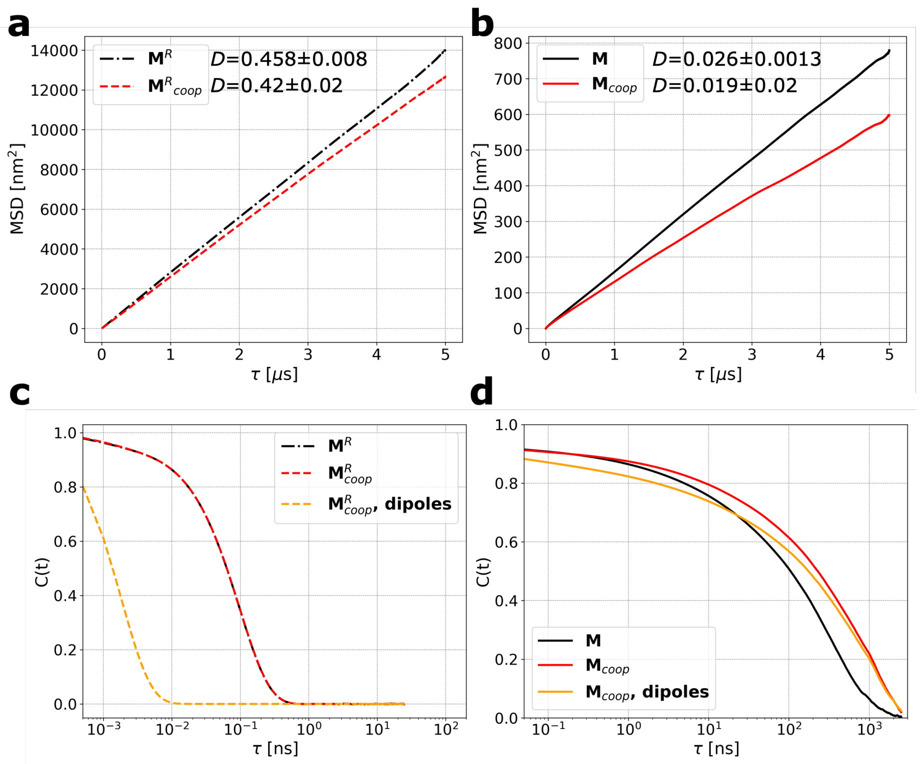

Figure S10: Diffusion and rotation dynamics at equilibrium. (a) mean-squared-displacement calculation for the $\mathbf{M}^R$ and $\mathbf{M}^R_{\text{coop}}$ systems, equivalent to $\mathbf{M}$ and $\mathbf{M}_{\text{coop}}$ but free from attractive (Coulomb or LJ) interactions, i.e. containing only free monomers. The diffusivities obtained by linear fitting are indicated besides the legend. (b) mean-squared-displacement calculation for the $\mathbf{M}$ and $\mathbf{M}_{\text{coop}}$ systems at equilibrium. The diffusivities obtained by linear fitting are indicated besides the legend. (c) Rotational correlation function of the $\mathbf{M}^R$ and $\mathbf{M}^R_{\text{coop}}$ monomers and of the dipole vectors of $\mathbf{M}^R_{\text{coop}}$. (d) Rotational correlation function of the $\mathbf{M}$ and $\mathbf{M}_{\text{coop}}$ monomers and of the dipole vectors of $\mathbf{M}_{\text{coop}}$.

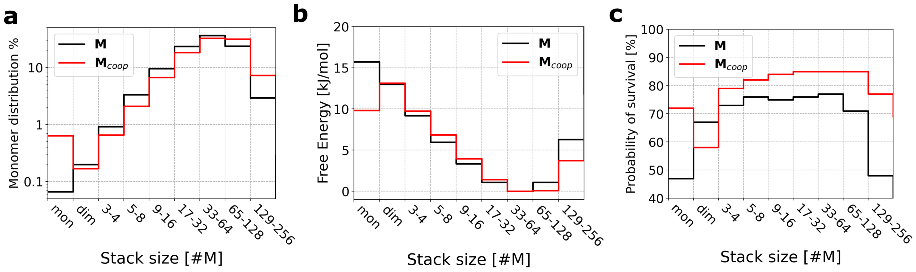

Figure S11: Cooperativity of $\mathbf{M}_{\text{coop}}$. (a) Distribution of monomers (in %) among the different assembled states, for the $\mathbf{M}$ (black) and $\mathbf{M}_{\text{coop}}$ (red) models. The $\mathbf{M}_{\text{coop}}$ exhibits a bimodal distribution associated to cooperative polymerization. (b) Free energy associated to how monomers populate the different aggregate states. The distributions in (a) indicate the probability P_{size} that a monomer is found in an aggregate state at the equilibrium. The free-energy can be computed as $F = -k_{\text{B}}T \ln(P_{\text{size}}/\max(P_{\text{size}}))$, where the reference value is the most populated state. The local minimum at the monomeric state of $\mathbf{M}_{\text{coop}}$ system is a signature of cooperativity. (c) Survival probability (in %) of a monomer in an aggregate state (with $\Delta\tau$=15 ns). This number correspond to the diagonal of the matrices in Fig. 2b of the main text. The panel underlines how the persistence in time of the different aggregates qualitatively differs between the two models. The assembly sizes are grouped in log binary scale in all panels.

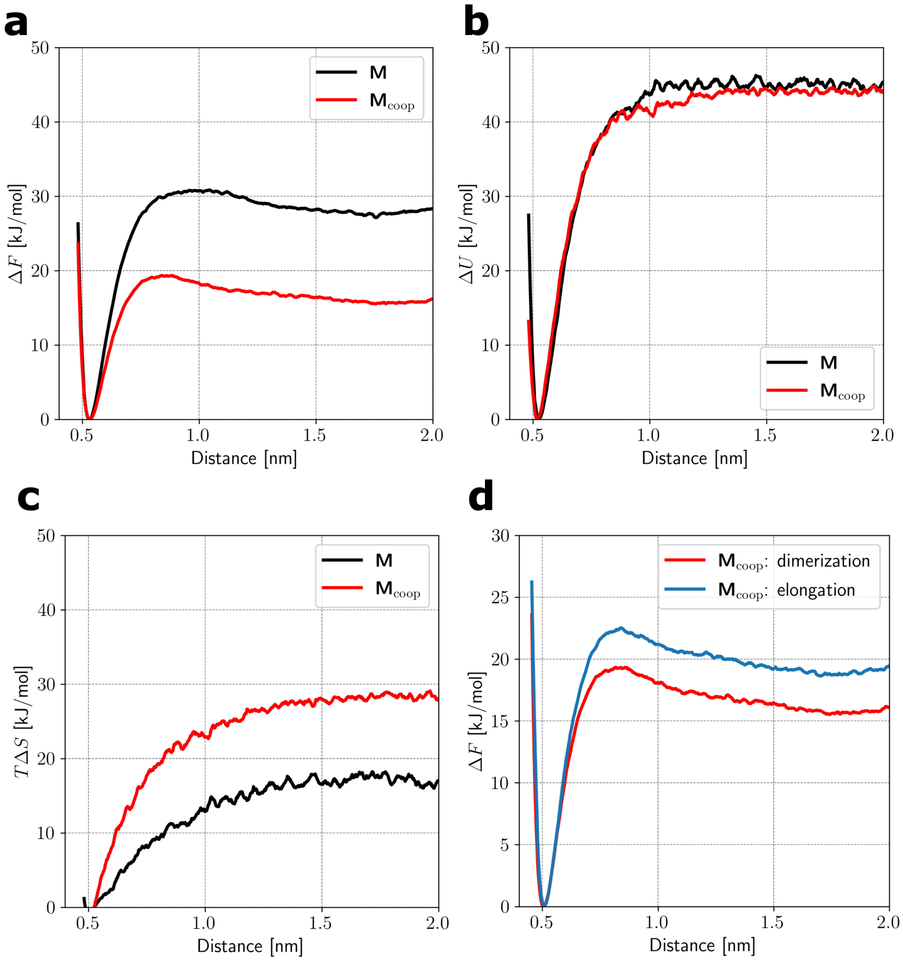

Figure S12: Dimerization process of $\mathbf{M}$ and $\mathbf{M}_{\mathrm{coop}}$ systems. (a) The free-energy of dimer formation is computed for the $\mathbf{M}$ and $\mathbf{M}_{\mathrm{coop}}$ systems via WT-Metadynamics simulations (see Methods), at the relevant density. (b) The energy of dimerization is calculated from the same WT-Metadynamics trajectories. (c) From (a) and (b) the entropic cost of dimerization can be computed, underlying that the entropic contribution is responsible for the lower propensity of the $\mathbf{M}_{\mathrm{coop}}$ system to form dimers. (d) The free energy of dimerization of $\mathbf{M}_{\mathrm{coop}}$ is compared to the free-energy of elongation, estimated by simulating the dimerization process of two $\mathbf{M}_{\mathrm{coop}}$ monomers, one of which has the dipole restrained perpendicular to the monomer plane via harmonic angular potentials, to mimic the dipole alignment of a fiber tip. The free-energy increase with respect to pure dimerization is a signature of cooperativity.
