# Supplementary material for: Non-trivial stimuli-responsive collective behaviours emerging from microscopic dynamic complexity in supramolecular polymer systems
Source: Nat Commun. 2025 May 30;16:5030. doi: 10.1038/s41467-025-60150-4 (PMC12125348; doi:10.1038/s41467-025-60150-4)
Supplement: Supplementary file 2 — Description of Additional Supplementary Files [file 41467_2025_60150_MOESM2_ESM.pdf]

Description of Additional Supplementary Files

File Name: Supplementary Movie 1
Description: Shows the molecular dynamics at supramolecular equilibrium of the M and Mcoop systems (N=500) in absence of C species (native) and upon addition of 100 C molecules. Each monomer is represented by a sphere located at its core. The color of the monomers is function of the size of the fibers they are part of. C molecules are not shown.
